# Lactate dehydrogenase to albumin ratio (LAR) is a novel predictor of fatal outcome in patients with SFTS: an observational study

**DOI:** 10.3389/fpubh.2024.1459712

**Published:** 2024-12-17

**Authors:** Tao Meng, Wenqian Ding, Dongmei Lv, Chenxu Wang, Yuanhong Xu

**Affiliations:** ^1^Department of Clinical Laboratory, The First Affiliated Hospital of Anhui Medical University, Hefei, China; ^2^Department of Pediatrics, The First Affiliated Hospital of Anhui Medical University, Hefei, China

**Keywords:** albumin, lactate dehydrogenase, severe fever and thrombocytopenia syndrome, LAR, predictor

## Abstract

**Background:**

Severe fever with thrombocytopenia syndrome (SFTS) is a serious infectious disease. This study explored the prognostic value of lactate dehydrogenase (LDH) to albumin (ALB) ratio (LAR) levels in fatal outcomes of the disease.

**Methods:**

Two-hundred and nine patients with SFTS were enrolled in this study. Based on the prognosis, patients were divided into survival and deceased groups. Laboratory metrics were compared by univariate Cox regression and multivariate Cox regression analyses. The prognostic risk factors for SFTS disease were discussed, and the receiver operator characteristic (ROC) curve and the Kaplan–Meier survival curve were plotted to analyze the predictive value of independent risk factors on disease prognosis.

**Results:**

A total of 209 patients with SFTS, including 152 in the survival group and 57 in the death group, were enrolled. The median age of 209 SFTS patients was 64 years. Three indicators, age, aspartate aminotransferase (AST), and LAR, were identified as predictors of mortality in patients with SFTS. The area under the ROC curve of LAR was the highest (0.835), followed by that of AST (0.794), and age (0.720). The Kaplan–Meier survival curve showed an increased case fatality rate, of >1.4691, in patients with LAR.

**Conclusion:**

Elevated LAR level on admission is an independent risk factor for fatal outcomes in patients with SFTS; this can help healthcare professionals identify patients with SFTS having a high risk of fatal outcomes.

## 1 Introduction

Severe fever with thrombocytopenia syndrome (SFTS) is a novel infectious disease due to severe fever caused by the Thrombocytopenia Syndrome Virus (SFTSV), of the genus *Sandfly virus* in the *Bunyaviridae* family, with ticks as its vector (1). This condition was first discovered in central and northeastern China in 2009 (2); subsequently, SFTS in South Korea (3), Japan (4), Vietnam (5), and other countries was also discovered. The primary clinical features of the disease are acute fever, thrombocytopenia, fatigue, and leukopenia. SFTSV infection mainly causes damage to the liver (6, 7), kidneys (8), and heart (9). Even in some severe cases, multiple organ failure can occur, ultimately leading to the death of the patient (10) with a case fatality rate of up to 30% (11). However, until now, no clinically effective antiviral drugs have been approved for SFTS, and the pathogenesis remains unclear; hence, early detection and intervention are extremely important for prognosis.

Lactate dehydrogenase (LDH) is a glycolytic enzyme generally included in myocardial enzyme profiles and liver function tests. The level of LDH has been found to be associated with the prognosis of many diseases, and their levels in the serum increase with disease severity. Studies have shown that LDH predicts poor outcomes in people with Coronavirus disease 2019 (COVID-19) and diabetic nephropathy (12, 13). According to reports, albumin (ALB) is associated with inflammation and is a powerful indicator of infection prognosis (14).

The LDH to ALB ratio (LAR) is a novel biomarker proven to be associated with the prognosis of pneumonia (15) and lower respiratory tract infections in emergency departments (16), which can improve the accuracy of disease diagnosis. However, there are no reports on whether the LDH/ALB ratio can be used as a prognostic factor for patients with SFTS. Therefore, this retrospective study aimed to explore whether serum LAR levels at admission can assist in evaluating the value of independent biomarkers for mortality in patients with SFTS.

## 2 Patients and methods

### 2.1 Study design and patient enrollment

**Figure 1:**
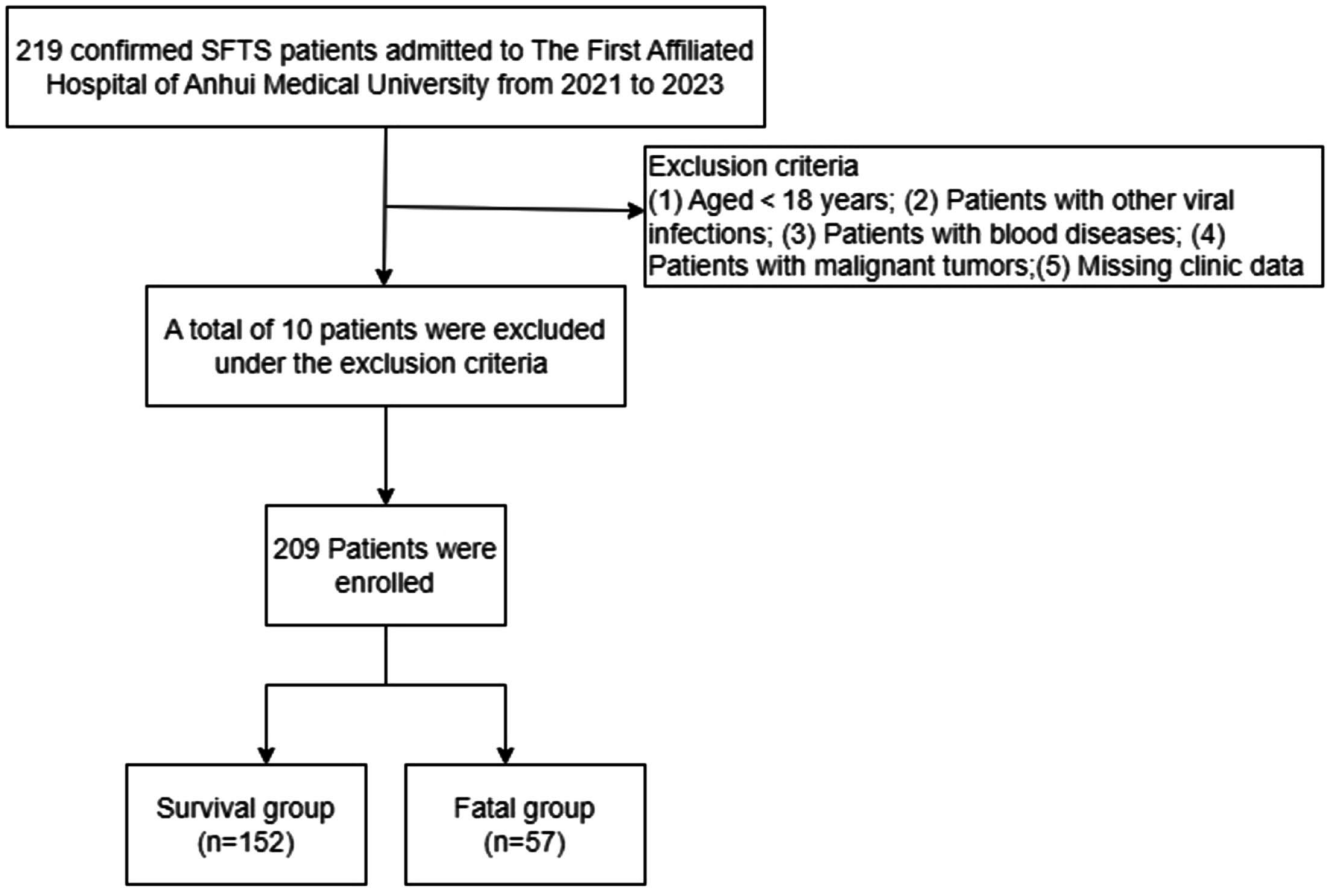
Schematic overview of the study design. SFTS, Severe fever with thrombocytopenia syndrome.

This study was proposed following the principles of the Declaration of Helsinki and approved by the First Affiliated Hospital, Anhui Medical University. In one retrospective analysis, 219 patients were included with SFTS confirmed at the First Affiliated Hospital, Anhui Medical University in China from January 2021 to September 2023. This hospital is in Hefei, Anhui Province, one of the most severely affected cities in central region of China. To improve the accuracy of the study, all patients were required to the fulfil the following diagnostic criteria: (1) acute fever with a temperature > 37.5°C, (2) thrombocytopenia (platelet count (PLT) < 100*10^9^/L), (3) serum nucleic acid detection or isolation of SFTSV (new Bunya virus). A total of 10 patients met the exclusion criteria: (1) younger than 18 years of age, (2) infection of other viruses, including hantavirus, Epstein–Barr virus, etc., (3) presence of a blood disease, (4) history of malignant tumor, and (5) patients with missing data (Figure 1).

### 2.2 Data collection

The medical records of all inpatients were kept in the medical record system of the hospital to ensure the authenticity of the data. A trained group of researchers collected relevant information and entered in an EXCEL form. The maximum value was taken for those greater than the upper limit in laboratory tests and values less than the lower bound were considered the lowest value. Baseline information included age, sex, duration from fever to hospital admission, length of hospitalization, history of smoking, history of alcohol, and a history of diabetes. Clinical symptoms included skin rash, chills, abdominal pain, diarrhea, nausea, vomiting, muscle aches, convulsions, and neurologic symptoms. Blood samples were collected the same day or the next day post-admission. Laboratory tests included white blood cell (WBC) count, neutrophil (NEU) count, lymphocyte (LYM) count, PLT, red blood cell (RBC) count, hemoglobin (HB), activated partial thromboplastin time (APTT), prothrombin time (PT), alanine aminotransferase (ALT), AST, creatine kinase (CK), LDH, ALB, total protein (TP), and C-reactive protein (CRP). The accuracy of the data was reviewed by another group of researchers. For patients with missing information, it was important to standardize the addition or counsel their families. Patients who discontinued treatment or were discharged for personal reasons were followed for 40 days after the start of admission. Death or hospital discharge were the endpoint of this study.

### 2.3 Statistical analysis

For continuous variables, data conforming to the normal distribution were represented by the mean ± standard deviation (SD), otherwise by median (M) and interquartile range (IQR). The categorical variables were represented by frequency and proportion. Differences between the two groups were analyzed by Student’s *t*-tests, Chi-square test, or nonparametric tests. The risk factors for patients with SFTS were obtained by first examining the relationships between all independent and dependent variables applying univariate Cox analysis, followed by multifactorial Cox analysis. The logarithmic transformations were performed on laboratory indicators (LAR, ALT, AST, and LDH) with large numerical variables in regression analysis. The accuracy of LAR prediction was evaluated by determining the area under the receiver operating characteristic (ROC) curve. The cumulative risk of death at LAR low and LAR high was assessed by Kaplan–Meier survival analysis, and the significance of the difference was tested using logarithmic rank. All data and graphs were statistically analyzed and produced using SPSS 27.0 statistical software, GraphPad prism 9 and R 4.3.0. A two-sided *p* < 0.05 was considered statistically significant.

## 3 Results

### 3.1 Demographic and clinical characteristics of patients with severe fever with thrombocytopenia syndrome

Two-hundred nine patients with confirmed SFTS were enrolled, including 152 survivors and 57 deaths, with a mortality rate of 27.3%. Patients were classified into a survival group and a deceased group based on prognosis. The median age of patients in the death group was 70.0 (64.0–74.5) years, which was significantly higher than that of the patients in the survival group, which was 61.5 (53.0–68.0) years (*p* < 0.001). Compared with the survival group, the median hospitalization time of 4 (2-8) days of the deceased group was shorter, but there were no significant differences in gender, days from fever to hospital admission, and history of smoking, drinking, and diabetes between the two groups. Clinical physical examination revealed a higher incidence of neurological symptoms in the deceased group (24/57, 42.1%) (Table 1). In terms of laboratory parameters, PLT and LYM in both groups were below the lower cut-off limit of the health (Table 2). The decline in PLT was more pronounced in the dead group. In addition to hematological tests, we observed elevation in serum enzyme values, including ALT, AST, and LDH. The levels of CRP also increased, and the PT time was also prolonged. Compared to the survival group, TP and ALB levels were significantly reduced in patients in the deceased group.

**Table 1:** Demographics and clinical characteristics of patients with SFTS.

Variables	Total (*n* = 209)	Survival (*n* = 152)	Fatal (*n* = 57)	*P* ^a^
Male, *n* (%)	88 (42.1)	64 (42.1)	24 (42.1)	1.000
Age (years), median (IQR)	64.0 (54.0–70.0)	61.5 (53.0–68.0)	70.0 (64.0–74.5)	<0.001
Time from onset to admission (days), median (IQR)	5 (3–7)	5 (3–7)	4 (3–7)	0.332
Hospitalization (days), median (IQR)	9 (6–12)	10 (8–13)	4 (2–8)	<0.001
Preexisting condition, *n* (%)				
History of smoking	20 (9.6)	12 (7.9)	8 (14.0)	0.179
History of alcohol	23 (11.0)	15 (9.9)	8 (14.0)	0.391
Diabetes	30 (14.4)	24 (15.8)	6 (10.5)	0.334
Clinical Symptoms, *n* (%)				
Skin rash	19 (9.1)	13 (8.6)	6 (10.5)	0.658
Chills	83 (39.7)	61 (40.1)	22 (38.6)	0.840
Diarrhea	96 (45.9)	69 (45.4)	27 (47.4)	0.799
Nausea	67 (32.1)	50 (32.9)	17 (29.8)	0.672
Vomiting	49 (23.4)	37 (24.3)	12 (21.1)	0.617
Muscle soreness	68 (32.5)	51 (33.6)	17 (29.8)	0.608
Convulsion	13 (6.2)	7 (4.6)	6 (10.5)	0.209
Neurological Symptoms	47 (22.5)	23 (15.1)	24 (42.1)	<0.001

SFTS, severe fever with thrombocytopenia syndrome; IQR, interquartile range. Data were expressed as median (IQR) or count (percentage, %) and were compared using the Chi-square test or Mann Whitney U test.

a P, comparing between the group of survival and the group of fatal.

**Table 2:** Laboratory results of patients with SFTS at admission.

Parameters (Reference range)	Total (*n* = 209)	Survival (*n* = 152)	Fatal (*n* = 57)	*P*
WBC (3.5–9.5 × 10^9^ /L)	2.72 (1.66–4.54)	2.72 (1.68–4.76)	2.66 (1.65–3.63)	0.343
NEU (1.8–6.3 × 10^9^ /L)	1.51 (0.95–2.81)	1.46 (0.95–2.98)	1.71 (0.92–2.57)	0.814
LYM (1.1–3.2 × 10^9^ /L)	0.69 (0.44–1.08)	0.71 (0.46–1.17)	0.56 (0.43–0.82)	0.090
RBC (4.3–5.8 × 10^9^ /L)	4.39 ± 0.61	4.42 ± 0.63	4.32 ± 0.54	0.279
Hb (130–175 g/L)	131.6 ± 18.3	131.4 ± 19.1	132.2 ± 15.9	0.782
PLT (125–350 × 10^9^ /L)	50.00 (37.00–73.50)	58.00 (41.00–79.75)	39.00 (27.50–53.00)	<0.001
APTT (11–14.5 s)	50.1 (43.5–54.8)	49.1 (42.7–54.3)	51.7 (45.0–55.9)	0.129
PT (11–14.5 s)	13.4 (12.7–13.8)	13.2 (12.6–13.6)	13.5 (13.1–14.4)	0.001
ALT (7–40 U/L)	74.0 (47.0–113.0)	68.5 (42.3–94.5)	116.0 (66.0–205.0)	<0.001
AST (13–35 U/L)	164.0 (85.0–306.5)	123.0 (70.5–216.0)	320.0 (209.5–708.5)	<0.001
CK (18–198 U/L)	362.0 (153.0–698.0)	316.0 (126.3–695.8)	452.0 (235.5–714.0)	0.055
LDH (120–250 U/L)	852.0 (509.5–1386.5)	683.0(422.0–1023.8)	1635.0 (926.5–3325.5)	<0.001
ALB (40–55 g/L)	33.7 ± 5.2	34.5 ± 5.4	31.6 ± 4.1	<0.001
TP (60–83 g/L)	63.7 ± 7.1	64.5 ± 7.5	61.5 ± 5.6	0.008
CRP (5–10 mg/L)	4.5 (1.3–11.8)	3.4 (1.2–11.5)	8.6 (3.4–18.4)	0.001

WBC, white blood cell; LYM, lymphocyte; NEU, neutrophils; RBC, red blood count; Hb, hemoglobin; PLT, platelet; APTT, activated partial thromboplastin time; PT, prothrombin time; LDH, lactate dehydrogenase; TP, total protein; ALB, albumin; CK, creatine phosphokinase; CRP, C-reactive protein; ALT, alanine transaminase; AST, aspartate aminotransferase. Data were expressed as mean ± standard deviation or median (IQR) and were compared using the Wilcoxon rank-sum test, Student’s *t*-test or Fisher’s exact test.

The recommended drugs for the treatment of severe fever with thrombocytopenia syndrome (17) are intravenous immunoglobulin and ribavirin. According to Table 3, there was no significant difference between the survival group and the death group in terms of using ribavirin, Intravenous immunoglobulin, and glucocorticoid drugs. In addition, in the death group, patients are more likely to receive treatment with blood products (platelets).

**Table 3:** Clinical treatment of SFTS patients.

Treatment	Total (*n* = 209)	Survival (*n* = 152)	Fatal (*n* = 57)	*P*
Antiviral therapy				
Ribavirin	170 (81.3)	130 (85.5)	40 (70.2)	0.182
Blood product therapy				
Albumin	42 (20.1)	27 (17.8)	15 (28.8)	0.169
Platelet	49 (23.4)	30 (19.7)	19 (33.3)	0.039
IVIG	179 (85.6)	132 (86.8)	47 (82.5)	0.421
Glucocorticoids	57 (27.3)	40 (26.3)	17 (29.8)	0.612

IVIG, Intravenous immunoglobulin; SFTS, Severe fever with thrombocytopenia syndrome.

### 3.2 Independent risk factor for death in patients with severe fever with thrombocytopenia syndrome

As shown in Table 4, through univariate Cox regression analysis, PLT, ALB, TP, and Hb were found to be considerably lower, while PT, LDH, ALT, AST and LAR concentration were recorder comparatively higher. The above-mentioned variables are important factors that affect the prognosis of SFTS patients. Variables with significant differences (*p* < 0.05) in univariate Cox analysis were further utilized for multivariate Cox analysis. The age (*p* < 0.001), AST (*p* = 0.007), and LAR (*p* < 0.001) were independent risk factors for death in patients with SFTS.

**Table 4:** Risk factors associated with disease prognosis of patients with SFTS.

Parameters	Univariate		Multivariate	
	HR (95%CI)	*P*	HR (95% CI)	*P*
Demographics and baseline characters				
Age	1.066 (1.037–1.094)	<0.001	1.078 (1.045–1.112)	<0.001
Male	0.996 (0.589–1.686)	0.989		
History				
History of smoking	1.639 (0.776–3.462)	0.195		
History of alcohol	1,341 (0.635–2.832)	0.441		
Diabetes	0.667 (0.286–1.555)	0.349		
Laboratory findings				
WBC	0.942 (0.846–1.049)	0.277		
LYM	0.661 (0.407–1.072)	0.093		
NEU	0.964 (0.858–1.084)	0.544		
RBC	0.831 (0.551–1.255)	0.380		
Hb	1.003 (0.989–1.017)	0.665		
PLT	0.984 (0.974–0.995)	0.004		
APTT	1.005 (0.979–1.032)	0.724		
PT	1.332 (1.127–1.575)	0.001		
LDH	12.885 (7.304–22.732)	<0.001		
TP	0.952 (0.917–0.989)	0.012		
ALB	0.914 (0.869–0.961)	<0.001		
CK	1.735 (0.954–3.156)	0.071		
CRP	1.005 (0.996–1.014)	0.320		
ALT	5.149 (2.804–9.456)	<0.001		
AST	6.433 (3.786–10.929)	<0.001	3.035 (1.346–6.844)	0.007
LAR	12.512 (7.171–21.830)	<0.001	12.598 (5.596–28.364)	<0.001

95% CI, confidence interval; LAR, lactate dehydrogenase to albumin ratio. Data were expressed as HR (95%CI) and were compared using Univariate Cox regression and multivariate Cox regression.

### 3.3 Diagnostic efficacy of age, aspartate aminotransferase and lactate dehydrogenase-to-albumin ratio on clinical outcomes in patients with severe fever with thrombocytopenia syndrome

The value of laboratory test indicators in predicting the death outcome of patients was evaluated. For this, the indicators related to the lethal outcome in the multivariate Cox were included in the ROC curve analysis. The predictive performance of age, AST, and LAR on SFTS prognosis was evaluated by area under the curve (AUC; Table 5). The AUCs of age, AST, and LAR were, respectively, 0.720 (95% CI, 0.643–0.797, *p* < 0.001), 0.794 (95% CI, 0.720–0.867, *p* < 0.001), and 0.835 (95% CI, 0.773–0.896, *p* < 0.001). Compared to the above variables, LAR exhibited better predictive performance.

**Table 5:** Predictive value of Age, AST, LAR in predicting SFTS severity.

Parameters	AUC	Cut off values	Sensitivity	Specificity	95%CI	*P*
Age	0.720	63.5	0.772	0.572	0.643–0.797	<0.001
AST	0.794	2.3962	0.702	0.822	0.720–0.867	<0.001
LAR	0.835	1.4691	0.772	0.743	0.773–0.896	<0.001

The cut-off points were selected by maximizing the sum of sensitivity and specificity. AUC, area under the ROC curve; LAR, lactate dehydrogenase to albumin ratio; AST, aspartate aminotransferase; 95% CI, confidence interval; ROC, receiver operating characteristic; SFTS, severe fever with thrombocytopenia syndrome. Data were compared using ROC curve and Jordon index.

**Figure 2:**
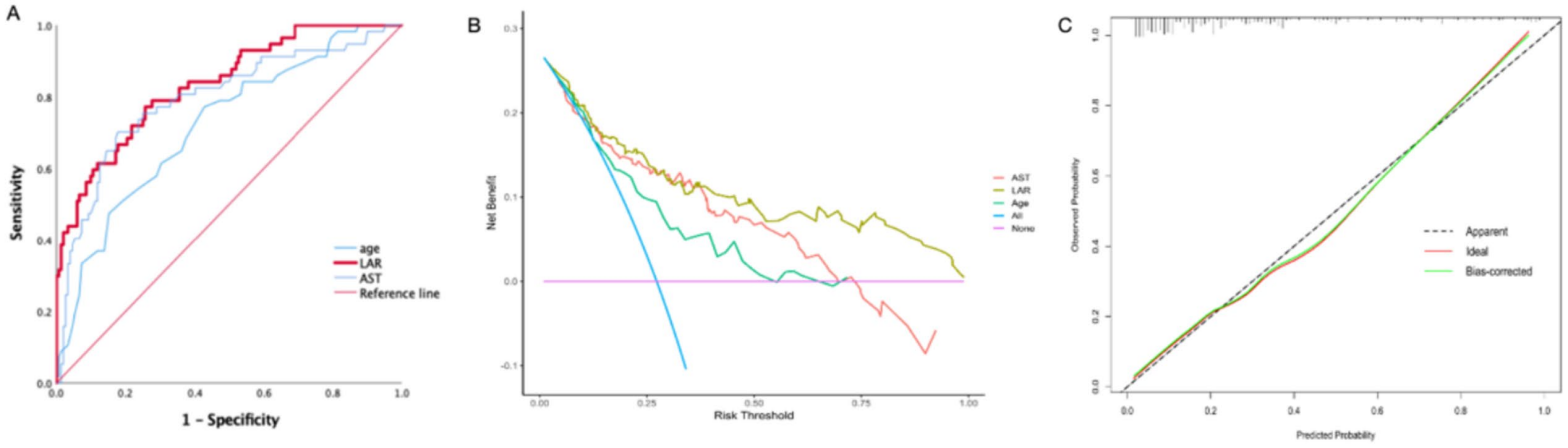
**(A)** ROC curves for evaluating the predictive ability of the LAR, AST, Age for SFTS severity at admission. **(B)** Decision curve analysis (DCA) of the LAR, AST, Age in SFTS patients. **(C)** Calibration curve for the LAR predicting fatal outcomes in SFTS patients. LAR, Lactate dehydrogenase to albumin ratio; SFTS, Severe fever with thrombocytopenia syndrome AUC, Area under curve; ROC: Receiver operating characteristic.

The sensitivity, specificity, and optimal cut-off value were determined according to the Youden index (Youden index = sensitivity + specificity −1). The value with the largest Youden index is chosen as the best cut-off value. The estimated cut-off value of LAR was 1.4691, with a sensitivity of 0.772 and a specificity of 0.743 (Figure 2A).

The decision curve analysis (DCA) showed excellent clinical efficacy of LAR in predicting adverse outcomes in SFTS patients (Figure 2B). Additionally, the calibration curve is near the ideal line, indicating that the predicted probability is very close to the actual probability (Figure 2C).

### 3.4 Prognostic value of lactate dehydrogenase-to-albumin ratio

**Figure 3:**
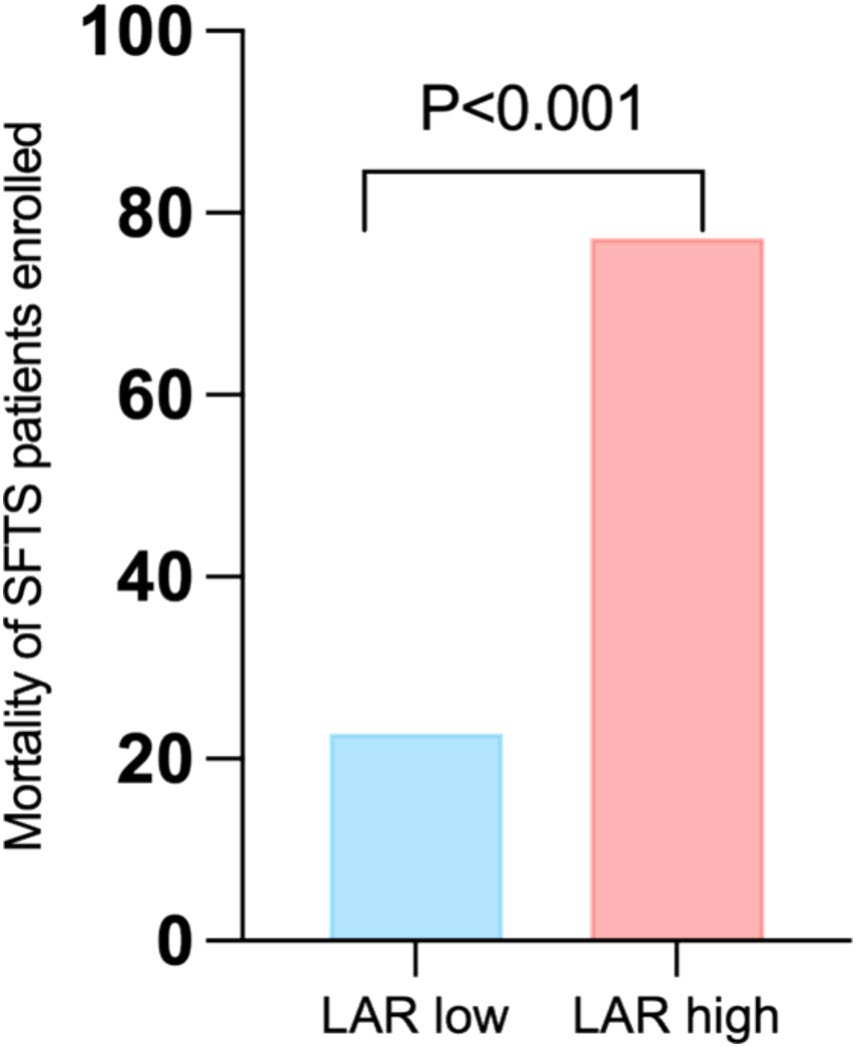
Comparison of fatality at different LAR levels. LAR, lactate dehydrogenase to albumin ratio. SFTS, Severe fever with thrombocytopenia syndrome.

Based on the cut-off value of LAR (1.4691), 209 patients with SFTS were categorized into two groups: the low LAR group (*n* = 126, 60.23%) and the high LAR group (*n* = 83, 39.71%). The fatality rate of patients with SFTS in the LAR high group was significantly higher than that in the LAR low group (10.32% vs. 53.01%, *p* < 0.001) (Figure 3).

**Figure 4:**
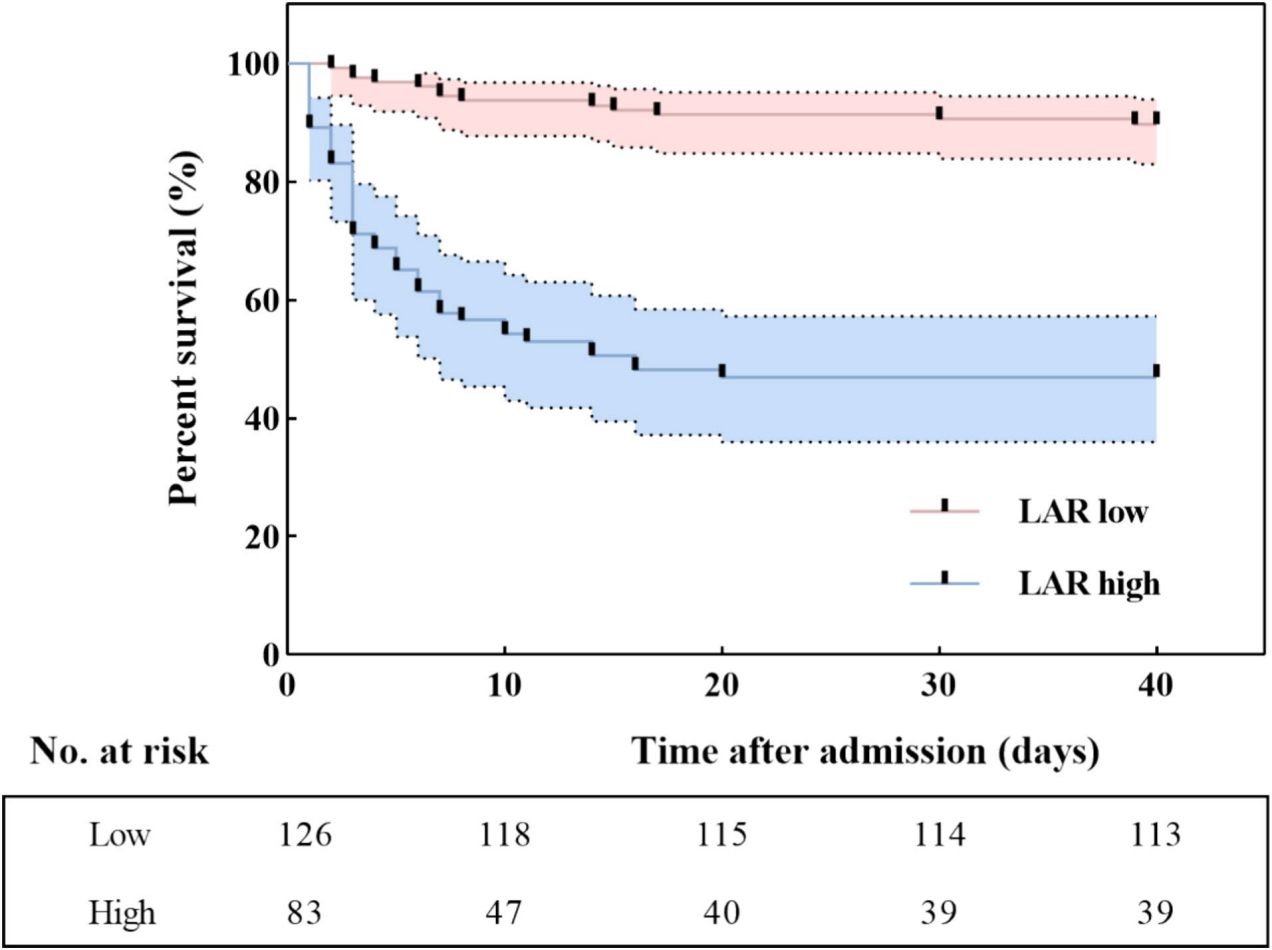
Kaplan–Meier survival curves according to the cut-off value of LAR. LAR, lactate dehydrogenase to albumin ratio.

Additionally, in the Kaplan–Meier survival curve analysis, patients with SFTS and having higher LARs had lower cumulative survival than those with lower LARs (Log-rank, *p* < 0.001) (Figure 4).

### 3.5 Correlation analysis between the LAR, ALB and AST

**Figure 5:**
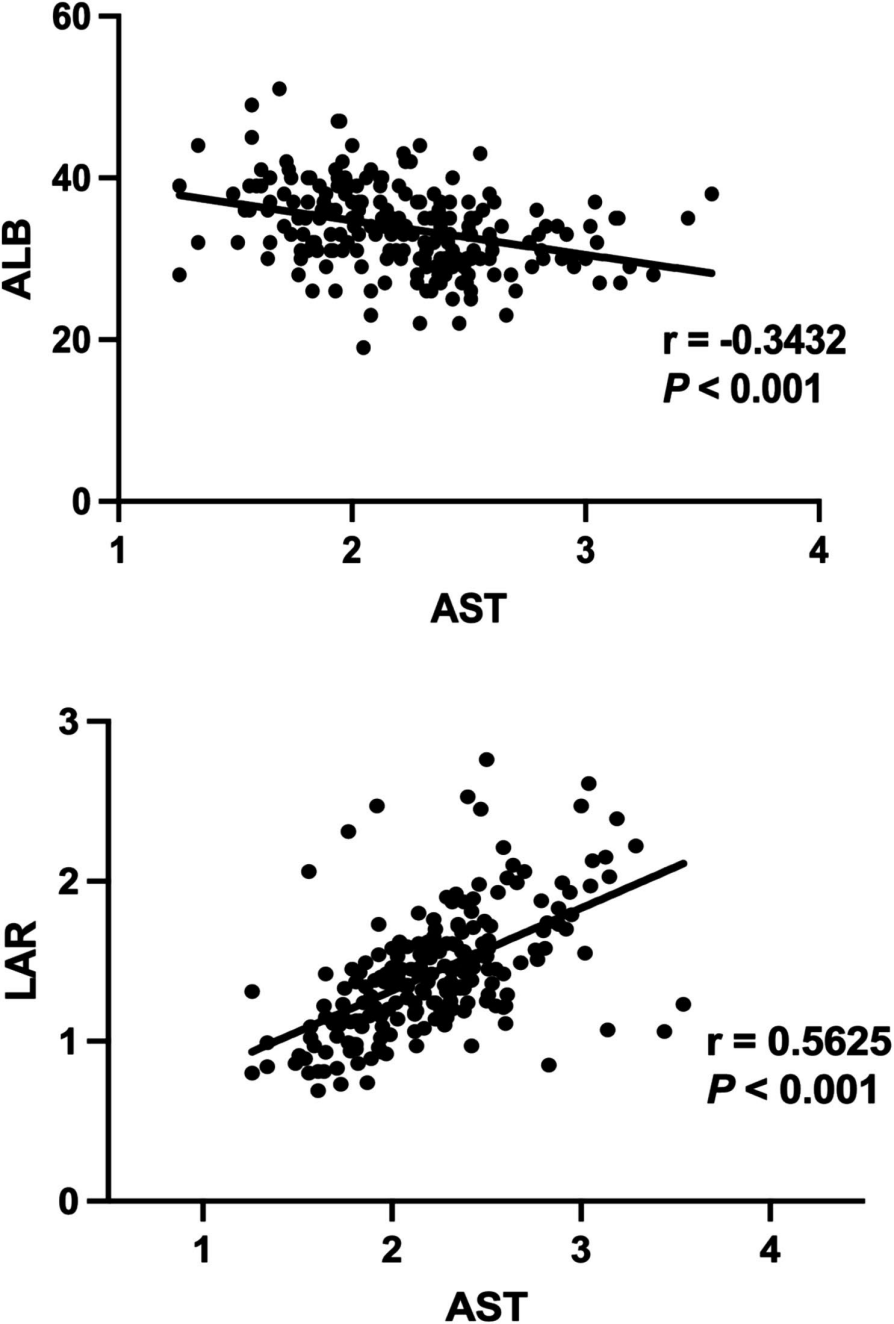
Correlation analysis between the AST and LAR, ALB.

We employed Spearman correlation test to analyze the relationship between LAR, ALB and AST variables. As shown Figure 5, it can be seen that a decrease in ALB synthesis is negatively correlated with liver injury (*p* < 0.001). There is a significant correlation (*p* < 0.001) between AST and LAR, with *r* = 0.5625, indicating a certain positive correlation between them.

## 4 Discussion

SFTS is caused by SFTSV infection, and the number of case reports have increased in recent years. The disease is characterized by rapid disease progression and high mortality; therefore, early recognition and early intervention are essential to improve the survival rate of patients. In this study, the medical data of 209 patients with SFTS were retrospectively studied to explore the risk factors affecting the disease prognosis. Multivariate Cox analysis showed that age, AST, and LAR varied between the two groups. Compared to other variables, the AUC of the LAR was relatively higher for the mortality rate of patients with SFTS. The cut-off value of LAR has strong potential in predicting the development and prognosis of SFTS.

In addition, we found that the older patients in the deceased group showed a statistical difference between the two groups, the same as previously reported (18–20). Older patients with weakened functions and immunity may have a corresponding increase in mortality. Research has shown that SFTSV can directly infect various cells, such as liver cells and immune cells, leading to cell death or organ dysfunction (21). In addition, viruses can activate the coagulation system, which may lead to disseminated intravascular coagulation (DIC) (22). Compared to the survival group, the levels of APTT, PT, TT, ALT, AST, LDH, and CK in the deceased group were higher, Indicating that liver damage and coagulation dysfunction in SFTS patients are associated with mortality.

LAR shows a good prognostic value in tumor diseases, reflecting inflammation and nutritional status in cancer. Likewise, the course of SFTS is often accompanied by systemic inflammatory responses and alterations in nutrient metabolism. Bacterial infection (23) or viral infection (24) cause elevated levels of LDH in serum. In an earlier prospective study, the LDH of patients with severe SFTS was significantly higher than that of non-severe patients (25). In addition, in the 105 patients enrolled in the First Affiliated Hospital of the University of Science and Technology of China, statistically significant differences in LDH levels were noted between deceased and survival groups (26). This may be related to the strong immune response triggered by SFTSV infection (27), and excessive inflammatory response leading to tissue damage; On the other hand, SFTSV infection causes endothelial cell damage and increases vascular permeability. This indicates that LDH can predict adverse outcomes in the case of SFTS infection (28–30). This is consistent with the results of our study. However, other studies have shown no statistically significant difference in serum LDH between the survival and deceased groups of patients with SFTS at admission (*p* = 0.06) (31). This inconsistency of research results may be attributed to different population characteristics. ALB is mainly produced by the liver and indicates the nutritional status of the body. Previous studies have shown that inflammatory bowel disease, cardiovascular disease, kidney injury, and other diseases can also cause abnormalities in ALB (32), and it is possible that inflammation can cause functional failure of multiple organs, which is a comprehensive manifestation of the body’s state. Hypoalbuminemia increases the mortality rate of patients with acute complications such as sepsis and shock (33) and the incidence of acute kidney injury (34, 35). There is a literature showing significant differences in ALB between patients with SFTS (36, 37). As shown in this study, ALB and eGFR significantly decreased in some cases. However, the use of a single indicator for evaluating diseases using LDH or ALB has limitations, as both are affected by multiple diseases, making the prognosis of SFTS patients difficult, and prone to misdiagnosis or missed diagnosis, especially in the early stages of infection. As shown in this study’s multivariate cox regression analysis, LAR is a better independent predictor for SFTS patients, while LDH is not. In the study by Feng et al., compared to LDH (*p* = 0.322), LAR (*p* = 0.038) can serve as a potential prognostic biomarker for identifying patients with esophageal squamous cell carcinoma without any auxiliary means (38). Nakazawa et al. (39) opined that an increase in serum LAR is a significant predictor of nivolumab resistance in patients with gastric cancer. Recently, Wu et al. (40) showed that LAR is associated with the prognosis and tumor staging of colorectal cancer but not with tumor differentiation in patients. In this study. we examined the association between LAR and the prognosis of patients with SFTS and found LAR as an important independent risk factor for SFTS.

To our knowledge, this is the first study to report the relationship between LAR and SFTS and is also significantly correlated with adverse outcomes in people with SFTS. Compared to the other indicators, this new biomarker showed the highest AUC. With elevated LAR, patients with early-stage SFTS may require close observation. LAR is an easily available and inexpensive biomarker; it can be determined by blood routine and biochemical routine and can also be a good choice for predicting the risk of fatal outcomes when the viral nucleic acid load cannot be determined or is difficult to detect. These results suggest that LAR could be widely used to predict and reduce high mortality.

However, the study has several limitations. First, this study is a single-center study, which may lead to a selective bias. Second, clinical laboratory tests are performed based on the status of the patient and the judgment of the attending physician. Therefore, the laboratory tests were not identical, for factors, such as interleukin-6 and procalcitonin, resulting in some missing data. Therefore, more standardized and systematic prospective studies are essential in the future to assess the clinical status of patients more accurately.

## 5 Conclusion

To conclude, according to our findings, LAR is an important combined biomarker for the diagnosis of SFTS, and its predictive performance is better than LDH and ALB singly. It helps clinical practitioners in the early identification of SFTS in patients with potentially fatal outcomes. Monitoring the inflammatory status as well as the nutritional status of the patient is important for the treatment of SFTS. Furthermore, future large-scale multicenter studies are needed to verify the utility of LAR as an objective biomarker for SFTS.

## Data Availability

The data analyzed in this study is subject to the following licenses/restrictions: Condensed anonymized data are available from the corresponding author on reasonable request. Requests to access these datasets should be directed to Yuanhong Xu, xyhong1964@163.com.
